# The Ocular Surface Microbiome in Homeostasis and Dysbiosis

**DOI:** 10.3390/microorganisms13091992

**Published:** 2025-08-27

**Authors:** Fiza Tariq, Navpreet K. Hehar, DeGaulle I. Chigbu

**Affiliations:** Pennsylvania College of Optometry, Drexel University, 8360 Old York Road, Elkins Park, PA 19027, USA; ft343@drexel.edu (F.T.); nh656@drexel.edu (N.K.H.)

**Keywords:** ocular surface microbiome, gut microbiome, dysbiosis, ocular surface immunity, 16S ribosomal RNA, probiotics

## Abstract

The ocular surface microbiome consists of microorganisms that play an important role in maintaining homeostasis and preventing disease from invading pathogens. Commensal microbes on the ocular surface interact with cells and molecules of the ocular surface immune system to promote immune tolerance to the normal flora of the ocular surface and facilitate immune protection against invading pathogenic microbes, which allows for a disease-free ocular surface. Various factors can impact the composition, distribution, and diversity of the ocular surface microbiome, including age, gender, disease state, antibiotic treatment, and contact lens use. In addition, there is no cohesive consensus on the species that make up the ocular surface microbes. There is, however, thorough research present on other similar mucosal membranes, such as the gut and oral mucosa, that share similarities with the ocular mucosa. Exploring the relationship of different mucosae allows us to explore treatment options for common ocular diseases such as dry eye syndrome. This review highlights studies that define the ocular surface microbiome, its diversity and composition, host–immune interactions at the ocular surface, factors that cause dysbiosis of the ocular surface microbiome, the impact of dysbiosis on the ocular surface microbiome, and microbiome-based therapy.

## 1. Introduction

Microorganisms exist throughout the human body, from the gut to the skin to the oral mucosa, and they together constitute the human microbiome [1,2]. Microbiota is defined as microbial species that are present in majority of the tested individuals at a particular location [3]. The largest microbial community is in the gut, where the number of microorganisms is comparable to the number of cells in the human body [4]. These microbes co-exist, thrive on healthy individuals, and play a vital role in maintaining immunity and fighting pathogens [2,3]. A team of bacteria, viruses, and fungi works in conjunction with the immune system of the ocular surface to maintain a disease-free environment [2].

Similarly, the ocular surface microbiome (OSM) has unique characteristics that play an important role in protecting the eyes from external factors [1]. The ocular surface is constantly exposed to the outside environment and thus has mechanisms in place to reduce the number of invading pathogenic microorganisms [5]. Mechanisms include blinking to replenish the tear film and the components of the ocular surface mucosal immune system, such as epithelial cells, antimicrobial molecules, and immune cells [6,7,8]. In addition to these defense mechanisms, the ocular surface microbiome maintains the natural homeostasis by encouraging probiotic and anti-inflammatory resident microbial bacteria that help fight off pathogenic bacteria [6]. One of those mechanisms includes the ocular surface epithelial cells, which selectively respond to pathogenic microbes through pro-inflammatory cytokines release and, in contrast, are tolerant to the ocular surface microbiome, consequently supporting the growth of a microbiota [5]. Moreover, commensal bacteria use competition to inhibit the growth of pathogenic bacteria and stop infection and inflammation [9]. As such, the interaction between commensal bacteria and the ocular surface mucosal immunity is essential for maintaining the homeostasis of the ocular surface [5].

The ocular surface microbiome is best defined by resident bacteria that do not cause inflammation or infection and live in harmony with the surrounding environment [4,10]. These bacteria help maintain ocular surface homeostasis by inhibiting the growth of pathogenic bacteria and contributing to immunologic tolerance in conjunction with the surrounding structures [11]. Additionally, ocular surface bacterial microbiome produces bacteriocins that possess antimicrobial activity that inhibits the growth of pathogenic microbes at the ocular surface [12].

The exact composition of the ocular surface microbiome has been challenging to study because it can be impacted by many extraneous factors such as age, gender, genetics, diet, and environment [4,13,14]. Moreover, active disease processes may cause chronic inflammation, and any topical medications applied to the ocular surface could impact the diversity and composition of the ocular surface microbiome [4,9,15]. Any alteration to the diversity and composition of the ocular surface microbiome can disrupt the homeostasis of the ocular surface, resulting in dysregulation of the ocular surface immune system [15].

Technology has advanced significantly in aiding the identification of the OSM, with the first tools being culture-dependent methods for identifying ocular surface organisms. However, next-generation sequencing techniques (16S ribosomal RNA, Shotgun metagenomics, and RNA-based metagenomic deep sequencing) have made it possible to have an enhanced characterization of the ocular surface microbiome compared to culture-based methodologies of characterizing the ocular surface microbiome [8,15]. Even with advancements such as genetic analysis and conjunctival swabs, the hunt for a “signature” OSM remains challenging to conclude [16].

In this review, we will highlight the difference in composition of the ocular surface microbiome based on culture-dependent and culture-independent methods. It will also address the relationship between the ocular surface mucosal immune system and the ocular surface microbiome. This review will also highlight the impact of ocular surface disease and environmental factors, such as antibiotic use and contact lens wear, on the diversity and composition of the ocular surface microbiome. Moreover, the impact of gut dysbiosis on ocular homeostasis and its role in the development of ocular surface disease will be reviewed.

## 2. Ocular Surface Microbiome Compared to Other Mucosal Microbiomes

The human body is composed of a large community of microorganisms on the epithelial and skin surfaces, including the ocular surface, urinary tract, and digestive and respiratory tracts [17]. These microbiotas include bacteria, viruses, fungi, and archaea [18]. Genetics, environmental, and lifestyle factors, including diet and geographic location, can significantly impact this microbe population [19,20,21].

The gut microbiome is a vast and diverse ecosystem of microorganisms [22]. It is associated with various functions that benefit the human body, including detoxification, immune system regulation, and protection against pathogens [22,23]. Accounting for about 70% of the bacterial composition, the major phyla in the gut bacteria include Firmicutes, Bacteroidetes, and Actinobacteria [24]. Another key area of rich microbial diversity is the oral cavity, with common phyla including Actinobacteria, Bacteroidetes, Firmicutes, Fusobacteria, Proteobacteria, and Spirochaetes [25,26].

The symbiotic evolution of the commensal bacteria and the host environment is mutually beneficial, as the gut provides nourishment and a reproduction site [27]. The gut microbiota, on the other hand, plays a crucial role in breaking down food, absorbing nutrients, and synthesizing proteins during immune modulation [28]. New research is indicating the important role the gut microbiome plays in the pathogenesis and progression of several extraintestinal disorders, including ocular diseases [29]. The gut–eye axis described in the literature highlights the close relationship between the ocular surface and the gut microbiome [30]. The gut microbiome maintains homeostasis by releasing short-chain fatty acids (SCFAs), bacteriocins, secondary bile acids, indoles, and polyamines [29]. In conditions with dysbiosis (imbalance), the overgrowth of pathogenic bacteria can lead to gut barrier disruption, metabolic endotoxemia, systemic inflammation, and retinal disease [7]. Many diseases, such as dry eye disease, age-related macular degeneration, diabetic retinopathy, uveitis, and glaucoma, have been associated with gut dysbiosis [31,32]. Research has also correlated oral microbiomes to various neurodegenerative diseases, including the progression of glaucoma, dry eye disease, and graft-versus-host disease [33,34].

It is important to understand the relationship between the gut and oral microbiome and ocular diseases, as it would aid in accurate diagnosis and provide individualized treatment and management options. Understanding the gut/ocular axis further may be useful in the development of new therapeutic interventions such as probiotics, prebiotics, symbiotics, and fecal microbiota transplantation.

## 3. Ocular Surface Microbiome Composition of a Healthy Ocular Surface

Age, geographic location, contact lens wear, diet, topical/oral antibiotic medications, ocular surgery, infections, or other ocular or systemic disorders can greatly vary the commensal ocular flora composition [16,35,36]. Studies have indicated that birth conducted vaginally vs. a cesarean section can have a dramatic effect on the abundance and type of ocular microorganisms [37].

Initial studies aiming to characterize the ocular surface microbiome used culture-dependent techniques that failed to identify the vast variety of species represented on the OSM [38]. Advances in technology have allowed us to utilize culturally independent methods, such as the sequencing of 16S ribosomal ribonucleic acid (rRNA) [39]. Studies that used the latter method allow the identification of entire microbial communities with their relative abundances in healthy and diseased cornea and conjunctiva, compared to previously used culture-based methods [40,41].

### 3.1. Technological Considerations for the Ocular Surface Microbiome

Conventional culture methods are limited by low microbial yield, limited diagnostic accuracy, and potentially long turnaround times for results [42]. A significant proportion of low-abundance microorganisms are difficult to culture and can often be missed by traditional culture methods [43].

The 16S rRNA gene sequencing technology is one of the most used methods in today’s world. Unlike the gut and nasal microbiome, the ocular surface microbiome has low microbial abundance. Amplicon sequencing addresses this by enabling the selection and amplification of bacterial marker genes through PCR before sequencing [44,45]. However, the amplification step can have limitations such as introducing biases on the initial template sequence, the DNA extraction method, and the number of PCR cycles used [8,46]. Moreover, 16S rRNA sequencing is primarily aimed at targeting bacteria, which can result in the omission of other kingdoms [46].

The term “next-generation sequencing” refers to modern techniques for rapid DNA sequencing methods [43,47]. Also known as high-throughput sequencing (HTS), they have been proven to be quicker and cheaper than older methods [43]. Metagenomic studies identify the microbes that form a community, but metatranscriptomics explore the diversity of genes within that community and observe how the genes interact with changes in their surrounding environment [47]. These modern technologies are revolutionizing the way we understand microbial communities, therefore bringing new information about both healthy and diseased ocular surfaces [48,49]. As this technology is increasingly used in laboratories, it can be expected to become the new standard for ocular surface microbiome analysis [48].

### 3.2. Bacteria

Traditional culture methods have been used for a long time to characterize various microbiomes [50]. Even though culture methods do help identify bacterial growth, they fail to comprehensively identify the complex network of bacterial species on the ocular surface [51,52]. Many studies show striking variation in the number of swabs that show positive bacterial growth, and even amongst those that do show growth, most of them identify only one microbial species [53].

The advent of molecular methods has allowed for a significantly improved and high-resolution method for detecting microbial species [54]. The 16S ribosomal RNA (rRNA) sequencing was first established in the 1980s and is a cornerstone for bacterial identification [55].

In healthy individuals’ ocular surface, culture-independent and dependent methods have long identified *Staphylococcus* on the species level as the most frequently identified organism on the ocular surface [3,56]. However, a recent systematic review of 11 published controlled cohorts using culture-independent methods (16S rRNA) found *Corynebacterium* as the most commonly identified species, followed by *Acinetobacter*, *Pseudomonas*, *Staphylococcus*, and *Cutibacterium* (Table 1) [57]. Other commonly found bacteria as part of the core commensal ocular microbiome include *Streptococcus* [5,56,58]. Unlike the previously mentioned bacteria, Gram-negative bacteria are less frequently found in healthy individuals. Gram-negative bacteria such as *Pseudomonas*, *Haemophilus*, and *Neisseria* species may be part of a non-diseased ocular surface microbiome, presenting minimal or no signs of inflammation or infection (Table 1) [59]. However, recent studies have also found high numbers of *Pseudomonas* species as part of the normal ocular flora, which goes against the previous findings of culture techniques [16,40,60].

Other recent studies have confirmed the bacterial diversity of the ocular surface microbiome and the challenges associated with its identification. Hezrog et al. [61] looked at swabs from the conjunctival eyelids of six participants. The study identified that the top most abundant bacterial species were *Staphylococcus epidermis*, *Cutibacterium acnes*, *Corynebacterium mastitidis*, and *Acinetobacter junii*. However, they identified major challenges associated with correctly identifying the composition of a lowly abundant ocular surface. They identified that the DNA extraction method and profiling tool can alter the composition of the ocular microbiome and present variation in the identified species. Thus, these factors must be considered in future studies aiming to characterize the ocular surface microbiome [61].

Another recent study by Ren et al. identified *Actinobacteria*, *Lactobacillus*, *Clostridium*, *Helicobacter*, and *Sphingomonas* as the core “interaction network” of the human conjunctival microbiome [62]. Their study further identifies how these microorganisms interact and work together to exemplify their antimicrobial and probiotic properties [62]. It has been shown that *Actinobacteria* were significantly reduced in the conjunctival microbiome of patients with microbial keratitis [63,64]. A specific genus of the *Actinobacteria*, *Corynebacterium*, has also been shown to aid in neutrophil recruitment and release antimicrobial peptides to boost immune protection at the ocular surface [65].

Another study by Zhou et al. [66] compared the microbiome of healthy patients vs. patients with clinical signs of trachoma. They identified that the microbiome varied significantly among the healthy participants based on the seasons the samples were collected and age, with more diversity noted in healthy and young patients. They found that compared to the microbiome of healthy patients, trachoma patients had a decrease in diversity and an increase in the number of *Corynebacterium* and *Streptococcus* species [66]. This study concludes that reduced bacterial diversity and changes in the commensal ocular flora are associated with trachomatous diseases [66].

In addition to different detection techniques, the method of data collection can significantly impact the test results. Chen and colleagues found that using tear test paper instead of conjunctival swabs for specimen collection led to differences in the detection of alpha and beta diversity of the ocular surface microbiome [67]. Alpha diversity was also higher in healthy subjects compared to those with ocular surface disease [68].

Huang et al., used high-throughput sequencing (HTS) technology to assess the OSM of 31 adult healthy human eyes without any prior history of ocular surgery or trauma, and the use of contact lenses [59]. Their advanced sequencing technology revealed a high microbial diversity. At the genus level, *Corynebacterium*, *Pseudomonas*, *Staphylococcus*, *Acinetobacter*, *Streptococcus*, *Millisia*, *Anaerococcus*, *Finegoldia*, *Simonsiella*, and *Veillonella* collectively comprised approximately 75 percent of the microbial community, possibly representing the core genera in the normal conjunctival microbiota [59]. Doan et al., compared traditional culture methods and HTS technology to identify the ocular surface microbiome in 89 healthy eyes with no history of contact lens use [41]. Consistent with previous findings, few organisms were identified using conventional culture methods [41,69]. However, HTS technology revealed substantially greater ocular microbial diversity than other techniques, identifying *Corynebacterium*, *Cutibacterium*, and coagulase-negative *Staphylococci* as the predominant microorganisms on the ocular surface [41].

### 3.3. Viruses

Following bacteria and fungi, viruses account for the next majority constituents of the OSM [8,70,71]. The ocular surface is home to native viruses which contribute to the OSM and play an important role in maintaining the homeostasis of the ocular surface [72]. Viruses such as adenoviruses and herpesvirus are well known for their pathogenic potential causing conditions such as conjunctivitis and keratitis. The presence of nonpathogenic viruses—collectively called a “virome”—may help modulate the immune response, specifically with bacteria-infecting viruses, called bacteriophages [8]. These bacteriophages help regulate and control the bacterial population, thus contributing to ocular surface homeostasis [8,71]. There are three populations of viruses that contribute to the OSM: Multiple Sclerosis-associated retrovirus (MSRV), Human Endogenous Retrovirus K (HERV-K), and Torque Teno Virus (TTV) (Table 1) [8,41]. Both MSRV and HEV-K are human endogenous retroviruses, whereas TTV is a single-stranded circular DNA anellovirus associated with seasonal hyperacute panuveitis and culture-negative endophthalmitis [4,41]. TTV is the most prevalent virus in the human virome and is believed to influence immune regulation through a TTV-specific CD8^+^T cell response, which results in reduced IFN-γ production and increased expression of the inhibitory NKG2A receptor [73]. While the clinical significance is unclear, emerging evidence indicates the virome plays an important role in the regulatory immune response [8,73].

### 3.4. Fungi

It is essential to note that the microbiome of the ocular surface encompasses a diverse range of bacteria, viruses, and fungi. The ocular surface microbiome consists of bacteria and fungi that are considered opportunistic pathogens. An imbalance in the ocular surface microbiome, referred to as dysbiosis, can facilitate the colonization of the ocular surface by pathogenic microbes [39], thereby increasing the susceptibility of the ocular surface to fungal infections, such as fungal keratitis [8,39,74]. *Malassezia*, *Rhodotorula*, *Davidiella*, *Aspergillus*, *Alternaria*, *Fusarium*, *Curvularia*, *Penicillium*, *Helminthosporium*, *Setosphaeria*, *Haematonectria*, *Candida*, *Choriomyces*, *Claedesporium*, *Saccharomyces*, and *Hormodendrum* are predominant genera of the ocular surface fungal microbiome or ocular surface mycobiome (Table 1) [30,75,76]. Using the next-generation sequencing technique, it was demonstrated that *Aspergillus*, *Setosphaeria*, *Malassezia*, and *Haematonectria* are the predominant genera of fungi present in the ocular surface fungal microbiome [75]. Shivaji and colleagues [76] demonstrated that Ascomycota and Basidiomycota are the most dominant phyla commonly found on the surface of the conjunctiva, and Parashanthi et al. [75] showed that *Aspergillus niger* and *Aspergillus flavus* are the predominant isolates of the ocular surface mycobiome. The 18S ribosomal RNA and internal transcribed spacer (ITS) gene amplicon are used in identifying fungi [77]. Phyla Ascomycota and Basidiomycota have been identified as the major phyla of the ocular surface fungal microbiome using ITS2 [8,78]. The culture-independent sequencing methods for characterizing the fungal microbiome overcome the challenges associated with culture-dependent methods, such as the inability to characterize fungi that require different growth conditions, fungi that have different growth rates, and fungi that are uncultivable [79]. Shivaji and colleagues compared the alpha diversity of the mycobiome of the conjunctiva on the basis of age and gender. They found that based on the Shannon diversity and operational taxonomic unit (OTU) number, there were no age-related changes in the conjunctival fungal flora [76]. Furthermore, the authors demonstrated that the alpha diversity of the ocular surface mycobiome in both eyes was similar, and that gender did not have an impact on the alpha diversity of the mycobiome of the ocular surface [76]. Thus, they demonstrated that alpha diversity of the conjunctival surface mycobiome was not influenced by gender, age, or eye being sampled. Shivaji and colleagues demonstrated that beta diversity of the fungal microbiome on the conjunctival surface was not influenced by age and the eye being sampled [76].

## 4. Ocular Surface Immunity and Its Interaction with the OSM

The cornea, meibomian gland, conjunctiva, limbus, and lacrimal gland are components of the ocular surface [80,81]. The ocular surface epithelial cells serve as an important physical barrier that constitutes a first-line defense against external threats at the ocular surface [5,82]. Epithelial cells of the ocular surface have pattern recognition receptors that detect the pathogen-associated molecular patterns (PAMP) of both commensal and pathogenic bacteria [15]. The binding of these PAMPs to pattern recognition receptors, such as toll-like receptors (TLRs), induces the activation of these TLRs to initiate a downstream cascade to generate the production of pro-inflammatory cytokines, chemokines, growth factors, and cell adhesion molecules that participate in the immune response at the ocular surface [15]. The epithelial cells tolerate commensal microbiota but induce an innate immune response to pathogenic bacteria [5]. Homeostasis of the ocular surface is a coordinated mechanism that involves the activity of the tear film mucin, secretory IgA, antimicrobial peptides in the tear film, and immune cells in the ocular surface. It is essential to to closely modulate the contact between the ocular surface epithelium and the microbiome of the ocular surface [4].

The conjunctiva plays an important role in immune surveillance at the ocular surface due to the presence of the epithelial layer, immune cells in the stroma, and intraepithelial lymphocytes. The conjunctival intraepithelial lymphocytes include MAIT (mucosal-associated invariant T) cells, natural killer (NK) cells, CD8^+^T cells, natural killer T cells (NKT cells), gamma delta T cells, and B1 cells [4,65,83,84]. Gamma delta T cells, MAIT cells, and NKT cells are nonconventional T cells that recognize non-peptide antigens independent of classic major histocompatibility complex (MHC) molecules [80]. Gamma delta T cells (γδT cells) are nonconventional T cells in the epithelium [80,85] that provide a first-line immune defense at epithelial tissues, including the ocular surface epithelium [86]. Gamma delta T cells play a major role in maintaining the homeostasis of the ocular surface and in promoting the epithelial barrier function of the cornea, which is accomplished by the action of IL-22 released by the gamma delta cells [86]. These immune cells hold a transitional position in innate and adaptive immunity [87]. MAIT cell is a nonconventional T cell with a semi-invariant T cell receptor (TCR) alpha chain, which consists of V_alpha_7.2-J_alpha_33 and is associated with the TCR beta chain (V_beta_2/V_beta_13) [88,89]. MAIT cells recognize microbial-derived riboflavin intermediates bound to the conserved major histocompatibility complex (MHC) Class 1-related molecule (MR1) [90]. Toll-like receptor 1 (TLR1), TLR2, and TLR6 are expressed on MAIT cells [88]. Antibacterial and antifungal host responses are mediated by MAIT cells in a TCR-dependent manner in response to riboflavin intermediates produced by bacteria and fungi. Antiviral host responses are mediated by MAIT cells in a TCR-independent manner because viruses do not express riboflavin intermediates [88,89,90]. Natural killer T (NKT) cells are nonconventional T cells that express TCR and NK receptors [91]. They promote an antibacterial, antiviral, and antifungal immune response at the ocular surface [91]. Type 1 NKT cells or invariant NKT cells express an invariant TCR, whereas type 2 NKT cell expresses a more diverse TCR repertoire [91]. MAIT cells, NKT cells, and gamma delta T cells are present in the healthy conjunctiva and can perform innate immune functions by providing immune protection at the ocular surface [83]. These lymphocytes can serve as a bridge between the normal flora of the ocular surface and the conjunctival-associated lymphoid tissue (CALT) [83]. The conjunctival goblet cells (CGCs) are located in the epithelium of the conjunctiva. The CGCs are linked to neighboring epithelial cells by tight junctions [82]. The CGCs produce muc5AC that maintains the integrity of the ocular surface epithelial barrier function. The CGCs produce defensins and other antimicrobial peptides that constitute the tear film defense, and as such, they act as a cellular component of the innate immune system [82]. The conjunctival goblet cells facilitate the clearance of pathogenic microbes [82]. Thus, the conjunctival goblet cells have a protective function at the ocular surface. Conjunctival goblet cells promote immunoregulation at the ocular surface due to their ability to secrete transforming growth factor beta (TGF-beta), an immunosuppressive cytokine that dampens the immune activation of antigen-presenting cells (APCs) in the ocular surface and makes them tolerogenic [92]. The tolerogenic dendritic cells migrate to the lymph nodes to activate naive T cells, which then become tolerogenic regulatory T cells (Treg cells) that arrive at the ocular surface epithelium to promote immunoregulation, thereby contributing to the homeostasis of the ocular surface [4]. TGF-beta promotes the differentiation and maintenance of regulatory T cells [81]. Thus, regulatory T cells and TGF-beta play a role in maintaining immune tolerance at the ocular surface [4,82]. The conjunctival immune system consists of the intraepithelial lymphocytes, subepithelial lymphoid follicles, lymphatic vessels, blood vessels, and immune cells found in the subepithelial layer of the conjunctiva [82]. The lacrimal drainage-associated lymphoid tissue (LDALT) and CALT provide immune surveillance to the ocular surface [92]. The conjunctival intraepithelial immune cells can interact with microbes independently of immune cells in the conjunctival lymphoid follicles [93]. CALT consists of a diffuse lymphoid tissue with immune cells and lymphoid follicles distributed in the stroma of the conjunctiva [80]. The lymphoid follicles in the conjunctiva contain APCs, B cells, and T cells [93,94]. The majority of the T cells in the CALT are γδT cells [15]. CALT promotes the induction of immune tolerance that prevents pro-inflammatory mediators from causing damage to the ocular surface [92]. Additionally, CALT induces and maintains tolerance to constituents of the ocular surface microbiome [81].

The cornea is an immune-privileged site due to the absence of blood vessels and lymphatic vessels, lack of MHC II or MHC Ia expression on corneal cells, the presence of immunosuppressive mediators, the expression of apoptosis-inducing molecules such as Fas Ligand (FasL) and programmed death-ligand-1 (PD-L1), and the role of regulatory T cells [80]. Soluble vascular endothelial growth factor receptor 1 (VEGFR1) and VEGFR3 sequester vascular endothelial growth factor A (VEGF-A) and VEGF-C, respectively, to limit the infiltration of immune cells into the cornea [81]. The corneal epithelium serves as the first line of defense by virtue of the presence of tight junctions that link the individual epithelial cells of the cornea to prevent access of environmental toxins and pathogens to the stroma of the cornea [92]. The tight junction of the corneal epithelium seals the interepithelial spaces on the corneal epithelium, thereby boosting the barrier protective function of the corneal epithelium [93]. The presence of an intact epithelial barrier, composed of tight junctions, facilitates the maintenance of ocular surface homeostasis and prevents the access of molecules and pathogens into the subepithelial layer of the cornea [92,93]. It is important to note that the corneal epithelium has a lower microbiome biomass than the conjunctiva; there is a greater proportion of the *Proteobacteria* phylum in the cornea [93]. Damage to the corneal epithelium results in a disruption of the innate immune system of the ocular surface due to loss of the epithelial barrier function, which is designed to prevent the entry of microbes into the subepithelial layer of the cornea [95]. A traumatized corneal epithelium upregulates the expression of mannosylated glycoprotein, which facilitates the attachment of fungi and protozoa to the traumatized corneal epithelium. The binding of these pathogens to the mannose glycoprotein leads to the development of microbial keratitis [80,96]. Traumatized corneal epithelial cells express CCL20 and ICAM-1, which facilitates the recruitment of γδT cells to the traumatized area, where γδT cells secrete IL-17 and IL-22 [80].

The toll-like receptor (TLR) is an innate immune sensor that detects the presence of damage-associated molecular patterns (DAMPs) and pathogen-associated molecular patterns (PAMPs) [5]. The TLRs of the ocular surface epithelium are capable of differentiating between normal flora and pathogenic microbes, promoting immune tolerance to the commensal flora, and facilitating the mounting of an immune response against potential pathogenic microorganisms [12,29]. TLR4 recognizes lipopolysaccharide (LPS), TLR2 recognizes lipoteichoic acid of Gram-positive bacteria, TLR2 recognizes peptidoglycan of both Gram-positive and Gram-negative bacteria, and TLR5 recognizes flagellin [5]. The ocular surface immune system tolerates the ocular surface microbiome because it maintains a state of immunosilence through the strategic intracellular location of TLR2, TLR4, and TLR5 at the level of the wing and basal epithelial cells of the corneal epithelium. These TLRs are not expressed on the superficial epithelium of the cornea [15,97,98,99]. Because of their intracellular location in the human corneal epithelium, TLR2, TLR4, and TLR5 can maintain an immune-silent state, preventing the initiation of an immune response against the normal flora of the ocular surface [5,98].

IgA-producing plasma cells in the lacrimal gland and conjunctiva produce large amounts of IgA that prevent pathogenic bacteria from binding to the ocular surface and facilitate the survival of commensal bacteria [100]. Interaction between the PAMP of commensal bacteria and TLR on dendritic cells is associated with the release of BAFF (B cell-activating factor of the tumor necrosis factor family) from dendritic cells [3]. BAFF binds to BAFF receptors on B cells in the conjunctiva to trigger the generation of IgA in a T cell-independent manner [3,101]. Additionally, TLR activation by PAMPs of pathogenic microbes can activate antigen-presenting cells, such as dendritic cells and macrophages, to release BAFF, thereby facilitating the T cell-independent production of IgA [15,102,103,104]. IgA-covered commensal bacteria in the ocular surface mucosa facilitate ocular surface homeostasis, and this enables the immune system to retain the ability to tolerate the commensal bacteria and mount a protective immune response to the presence of pathogenic bacteria [105]. The commensal microbiome maintains the ocular surface microbiome by facilitating the presence of secretory IgA in the tear film [3]. Thus, commensal bacteria covered with non-inflammatory IgA induce an immunoregulatory environment that supports the survival of commensal bacteria [105]. Secretory IgA in tears binds to pathogenic microbes, facilitating their removal from the ocular surface [92]. It is important to note that secretory IgA in the tear film facilitates the production of IL-10 to induce immune tolerance at the ocular surface [15]. IgA specific for *Staphylococcus intermedius* (a normal flora of the ocular surface) can cross-react with *Staphylococcus aureus*, reducing the colony size of *Staphylococcus aureus* on the ocular surface [15,102]. Commensal microbiome-induced secretion of IgA reinforces the immune tolerance to commensal bacteria and immune protection against nonself antigens [3].

**Figure 1:**
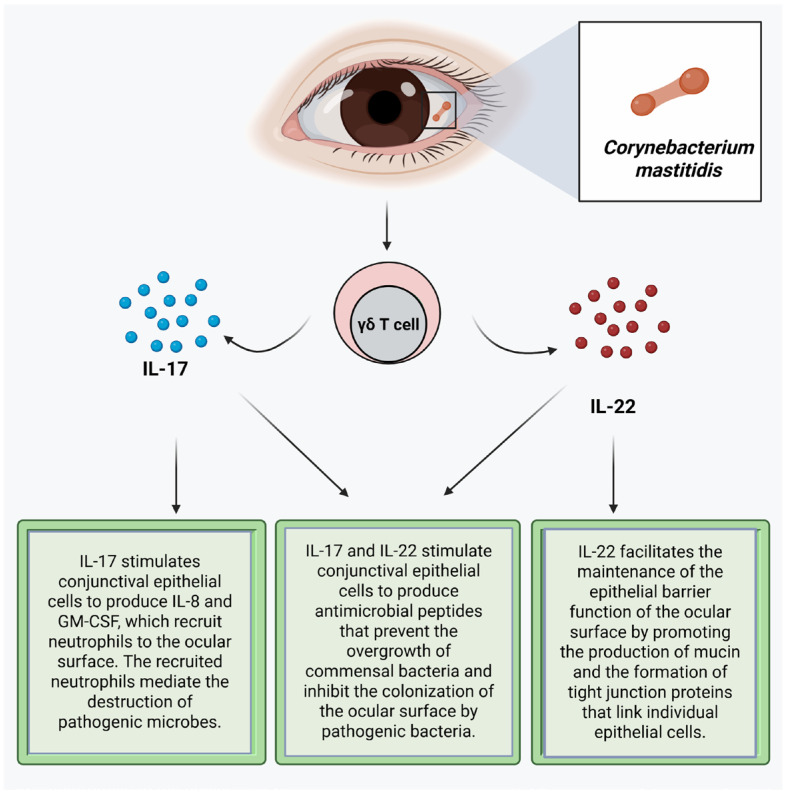
*Corynebacterium mastitidis* promotes resistance to pathogenic bacteria by inducing gamma delta T cells in the conjunctiva to secrete IL-17 and IL-22. Created with biorender.com.

*Corynebacterium mastitidis* is a nonpathogenic bacterium frequently found in the ocular surface microbiome [16,83,97,106]. This commensal bacterium promotes resistance to colonization of the conjunctiva by pathogenic bacteria by inducing gamma delta T cells in the conjunctiva to secrete IL-17 and IL-22 (Figure 1) [3]. IL-17 stimulates conjunctival epithelial cells to produce antimicrobial peptides, such as defensin, that prevent the overgrowth of commensal bacteria and inhibit the colonization of the ocular surface by pathogenic bacteria [5,65,92]. IL-17 stimulates the conjunctival epithelial cells to produce IL-8 and Granulocyte–Macrophage Colony-Stimulating Factor (GM-CSF), which recruit neutrophils to the ocular surface that mediate the destruction of pathogenic microbes through phagocytosis, [65,107,108,109] production of reactive oxygen species, and the release of neutrophil extracellular traps (NETs) [93]. These NETs aggregate epithelial cells and other neutrophils, where the serine proteases proteolytically degrade the aggregates [93]. IL-17 also promotes the regeneration or reestablishment of the epithelial barrier function by inducing the proliferation of epithelial stem cells and inducing the expression of tight junction proteins [107]. IL-22 stimulates the production of antimicrobial proteins from epithelial cells. It facilitates the homeostasis of the epithelial barrier, which helps maintain epithelial barrier function by promoting the production of mucin and the formation of tight junction proteins that link individual epithelial cells [110,111]. IL-22 stimulates the proliferation of corneal epithelial cells during the wound healing process to promote the maintenance of the homeostasis of the ocular surface. Thus, IL-22 promotes re-epithelialization of the cornea [80,86]. *Staphylococcus epidermidis* produces a serine protease that destroys the biofilm of *Staphylococcus aureus*, thereby exposing *Staphylococcus aureus* in the biofilm to destruction by the immune system [4,112]. *Staphylococcus epidermidis* also produces riboflavin metabolites, and as such, it can contribute to the priming of MAIT cells, thereby boosting the barrier immunity [113]. Commensal bacteria-induced immune responses facilitate the reinforcement of ocular surface immunity, limiting the increase in colony size of the commensal bacteria and preventing infection by pathogenic bacteria [4].

## 5. The Impact of Dysbiosis of the Ocular Surface Microbiome

### 5.1. Dry Eye Disease

Dry eye disease is associated with disruption and a change in the diversity of the ocular surface microbiome and gut microbiome [114,115]. Epithelial cells, dendritic cells, and innate lymphoid cells are key components of the innate immune system in the gut, which senses microbial antigens or metabolites to regulate the host–microbe interface in the gut [116]. T cells and B cells in the gut mediate adaptive immunity to ensure the maintenance of immune tolerance to gut commensals and mount a protective immunity against pathogenic microbes. IgA is the predominant antibody in the gut, and it is produced by B cells through both T cell-dependent and T cell-independent pathways [117].

The gut bacterial microbiome plays a vital role in the development of the immune system and in educating it to distinguish between harmful pathogenic microbes and commensal microbes. It provides defense against pathogens in the gut and enhances immunomodulation by SCFA-producing bacteria [12,118]. Changes in the composition of gut microbiota can result in a reduction in the level of SCFA-producing gut commensals and an imbalance between regulatory T cells (Treg cells) and Th17 cells [116]. The use of antibiotics, high-fat diets, chronic stress, and high sugar diets are major causes of gut dysbiosis. A high-fat diet stimulates the growth of *Bilophila wadsorthia*, a bile-tolerant bacterium that produces hydrogen sulfide, which in turn disrupts the gut barrier. An excessive sugar diet negatively impacts the gut microbiota by reducing the production of SCFA by commensal bacteria. Thus, high-fat and high-sugar diets have a negative impact on gut barrier function [12,119,120]. Cortisol, a stress hormone released under chronic stress conditions, disrupts the gut microbial balance by promoting the growth of opportunistic bacteria and impairing the survival of commensal bacteria. Cortisol can impair the gut immune system [12,121]. Antibiotics target both pathogenic and commensal bacteria in the gut, which in turn results in a reduction in the diversity of the gut microbiota [120,122,123].

The concept of the gut–eye axis refers to the role that gut microbiota play in modulating ocular surface immunity [12], and a disruption of the gut microbiota can lead to eye disorders due to immune dysregulation [115]. In the proposed model of the gut–eye axis, it has been suggested that changes in the gut microbiota can contribute to the development of dry eye disease [124]. The connection between gut microbiome dysbiosis and dry eye disease remains an area of active research, and it has been proposed that gut dysbiosis can induce dry eye disease through various mechanisms. Dendritic cells activated by microbiota in the setting of gut dysbiosis can migrate to the lacrimal gland, regional lymph node, and ocular surface, and induce the activation and differentiation of naive T cells into effector T cells that release pro-inflammatory cytokines in the ocular surface [116]. Another mechanism by which gut dysbiosis contributes to dry eye disease is the migration of gut-primed effector T cells and autoreactive B cell-derived autoantibodies to the ocular surface, leading to inflammation in this area [115,116]. Molecular mimicry is another mechanism by which antigens derived from microbes resident in the gut microenvironment of dysbiosis, with epitopes similar to those of Ro/SSA autoantigens, can cross-prime autoreactive CD4^+^T cells. These cells provide help to B cells, enabling them to produce anti-Ro/SSA autoantibodies that migrate to the lacrimal gland, causing inflammation that results in dry eye disease [116]. Gut dysbiosis disrupts the integrity of the gut barrier, leading to increased intestinal permeability and allowing bacteria and harmful substances, such as lipopolysaccharide (LPS), to pass from the gut into systemic circulation. The leakage of LPS into the systemic circulation provides LPS the opportunity to interact with TLR on immune cells, inducing them to become activated immune cells that release pro-inflammatory cytokines, culminating in inflammation and immune dysregulation that can contribute to the development of dry eye disease [12]. Additionally, gut dysbiosis reduces the production of SCFA [125]. Thus, gut dysbiosis can lead to a reduction in conjunctival goblet cell density, disruption of the corneal epithelial barrier function, decreased tear film secretion, and a reduction in tear breakup time. Gut dysbiosis is associated with an imbalance between Treg cells and Th17 cells as well as a decrease in SCFA-producing bacteria [116].

Willis and colleagues suggested that an increase in the abundance of microbes under closed-eye conditions can alter the T cell subpopulation on the ocular surface, leading to the development of dry eye disease. They further suggested that under closed-eye conditions, there is a high likelihood of dysregulation of ocular surface mucosal immunity, with the consequence of increasing microbial abundance [126]. In their study, Willis and colleagues demonstrated that altered microbial diversity is a hallmark of dry eye disease [126]. This is evident by a change in the level of Th17 cells due to perturbations of the ocular surface microbiome in dry eye conditions [126]. Sjogren’s syndrome dry eye (SSDE) in the setting of gut dysbiosis is associated with an increase in the level of type I interferons, TNF-alpha, IL-2, IL-7, interferon gamma, IL-17, Th1 cells, Th17 cells, B cells, and autoantibodies in the lacrimal gland. Th1 cells, Th17 cells, and autoreactive B cells are major players in the pathophysiology of SSDE [116]. It has been demonstrated that NSDE in this setting of gut dysbiosis is associated with increased levels of IL-1, IL-6, TNF-alpha, IFN-gamma, and IL-17. There is also an increase in the level of Th1 and Th17 cells. In NSDE, IL-17 induces epithelial cells of the ocular surface to secrete matrix metalloproteinase (MMP) that causes damage to the epithelium of the cornea and conjunctiva. Thus, an imbalance of Treg cells and Th17 cells on the ocular surface is a major player in the pathogenesis of NSDE in the setting of gut dysbiosis [116]. It has been shown that there is an increase in the level of Acinetobacter bacteria in both Sjogren’s syndrome dry eye (SSDE) and non-SSDE individuals, and individuals with SSDE have demonstrated an increase in the level of Corynebacterium [114]. This indicates that dry eye disease can significantly change the ocular surface microbiome, resulting in a consequential reduction in the diversity of the ocular surface microbiome [114,127]. Yunn Qi and colleagues [128] used 16S ribosomal RNA gene application sequencing to explore the difference in the diversity and composition of the ocular surface microbiome in individuals with dry eye with and without autoimmune diseases and the group found that there were significant differences in the composition of the ocular surface microbiome between individuals with autoimmune dry eye disease and individuals with non-autoimmune dry eye disease. Individuals with autoimmune dry eye disease had significant changes in the beta diversity of the ocular surface microbiome compared to individuals with non-autoimmune dry eye disease. The authors demonstrated an increase in the colony size of Corynebacterium in individuals with autoimmune dry eye disease [128,129]. Gupta [130] demonstrated an increase in the level of Streptococcus in individuals with non-autoimmune dry eye disease, whereas Kim [131] showed an increase in the level of Corynebacterium in individuals with autoimmune dry eye disease, such as SSDE, which implied that a reduction in the diversity of the ocular surface microbiome is commonly associated with SSDE. It has been demonstrated that individuals with autoimmune-associated dry eye disease and non-autoimmune dry eye disease have an increase in the level of Streptococcus and Corynebacterium [114]. As the severity of dry eye worsens, there is a further reduction in the diversity of the ocular surface microbiome, which in turn results in a more homogeneous bacterial ocular surface microbiome and a high likelihood of developing ocular surface infection due to the increase in the colony size of pathogenic bacteria [132].

Probiotics and prebiotics have health benefits in ameliorating several diseases associated with gut dysbiosis. It has been demonstrated that probiotics and prebiotics produce clinical benefits in individuals with dry eye syndrome [116]. Chisari and colleagues [133] showed that administering probiotics containing *Bifidobacterium lactis* and *Bifidobacterium bifidum* to individuals with dry eye syndrome results in an increase in tear film secretion and an increase in tear breakup time (TBUT). Chisari and colleagues demonstrated that individuals with dry eye syndrome experience a reduction in dry eye symptoms and an increase in the TBUT and tear secretion following the administration of probiotics containing *Saccharomyces boulardii* MUCL53837 and *Enterococcus faecium* LMGS 28935 [134].

### 5.2. Meibomian Gland Dysfunction

The meibomian glands are specialized sebaceous glands located in the upper and lower tarsal plates, responsible for secreting meibum, the lipid layer of the tear film. Proper function of these glands is essential for eye comfort and vision, whereas dysfunction can lead to dry eye disease and ocular inflammation. Altered meibum is the hallmark of meibomian gland dysfunction (MGD). The OSM contributes to maintaining ocular surface health by supporting microbial and immune homeostasis and modulating host immune responses to protect against pathogenic invasion [15,93]. Ocular surface microbiome changes can affect the meibomian glands leading to meibomian gland dysfunction [15,135,136]. Studies have shown increased bacterial species within the conjunctival sac in patients with MGD compared to healthy subjects [137,138]. Interestingly, there are also reports of comparable microbiomes within the conjunctival sac of patients with and without MGD [139,140]. Data surrounding the ocular microbiota in healthy individuals varies depending on the method of microbiome investigation [4,72]. Additionally, the composition of a healthy microbiome can be dictated by environment, age, and also by sex; therefore, there is no consensus on the composition of a core microbiome [4,141]. The most abundant genera on the ocular surface in healthy individuals includes *coagulase-negative Staphylococci* [59,71,139,141,142], *Cutibacterium* [40,41,66,71,141], *Corynebacterium* [40,41,54,59,66,71,139,141], *Staphylococcus* [41,66,71,139], *Streptococcus* [40,41,54,66,71,141], and *Pseudomonas* [40,59,141]. It has also been noted that bacteria of the *Corynebacterium* genus are commonly present in healthy conjunctival tissue [59,93,140].

Bacteria become attached to the tear film due to glycosaminoglycans (GAGs) and extracellular matrix proteins, where GAGs act as receptors for bacterial adhesion [4]. This cluster of bacterial microorganisms attaches to both each other and the ocular surface, forming a biofilm—an organized, multi-species community encased in an extracellular matrix, where bacteria can exchange nutrients and are protected from harmful agents such as antibiotics and the host’s immune system [4,141]. Bacterial biofilms can produce a variety of harmful substances, including enzymes which trigger inflammatory responses and increase the host’s susceptibility to infection [4,143]. When these biofilms form along the eyelid margin, they can induce chronic inflammation, manifesting as blepharitis and obstructing the meibomian glands. This obstruction disrupts the normal secretion of lipids essential for maintaining a stable tear film, ultimately contributing to the development of meibomian gland dysfunction [4]. Imbalances in the bacterial microbiota—known as dysbiosis—can promote the formation of bacterial biofilms within the conjunctival sac and lead to varying degrees of MGD and chronic inflammation [71,140]. Individuals with moderate MGD have been found to have the lowest concentration of *Staphylococcus*, whereas those with severe MGD had a higher concentration of *Cutibacterium acnes* [139]. Interestingly, in another study, those with severe MGD were found to have a higher concentration of Staphylococcus compared to the control group [140,144]. This same study also found a higher concentration of *Corynebacterium* in patients with moderate/severe MGD [144]. The abundance of *Staphylococcus* has been correlated with the severity of meibomian gland loss, where increased density is thought to lead to an increase in exotoxins and enzymes that disrupt the ocular surface [140]. Changes in the composition of the bacterial community within conjunctival tissue can lead to cytotoxicity and an increase in inflammatory components, which can contribute to the development of MGD [136,137,140,145].

### 5.3. Ocular Allergy

Allergic conjunctivitis (AC) is an inflammatory response of the conjunctiva in response to specific allergens [146]. The inflammatory response is mediated by allergen-induced crosslinking of IgE bound to FCepsilon receptor I (FCεRI) on primed conjunctival mast cells, which in turn become activated and, in response, lead to activated and degranulated mast cells releasing histamine, which causes the symptoms of itching, swelling, and redness [147,148,149]. The pathogenesis of allergic diseases is complicated and is influenced by various environmental and genetic factors [146]. Studies have shown that local microbial communities in other parts of the body, such as the gut, respiratory tract, and skin, may be associated with an increased risk of allergy [146,150]. The relationship between allergic conjunctivitis and the ocular microbiome remains poorly explored and needs to be further investigated to understand the key underlying mechanisms and provide effective treatment strategies [146]. Emerging research underscores the significance of ocular microbiome composition and suggests that sudden disruptions may contribute to the development of chronic inflammatory conditions, including ocular allergies [151].

The OSM is influenced by various external factors that impact the ocular surface’s health and, in turn, its composition. Vincenzo and his colleagues assessed the influence of the physiological, environmental, and lifestyle factors on the OSM. They extracted DNA using the 16S RNA gene sequencing method from 135 healthy individuals [141]. The study found that the highest number of differential microbial genera was found in individuals with allergies and highlighted that the microbiome plays a central role in balancing immune tolerance and activation, which contributes to the pathogenesis of conditions like atopic dermatitis [141,152]. Another study by Vishwakarma and colleagues assessed the ocular surface microbiome profile of patients with vernal keratoconjunctivitis and healthy subjects. They enrolled 30 patients with VKC and matched controls and assessed their tear film profile using polymerase chain reaction (PCR), traditional culture methods, and microscopy. *Staphylococcus aureus* was found to be the most common ocular surface flora and isolated in 70% of patients with VKC [153]. Even though the microbial diversity was similar, the bacterial load was higher in VKC patients [153,154]. Wang and colleagues also explored the microbial diversity in healthy subjects vs. patients diagnosed with allergic rhinoconjunctivitis (ARC) and allergic rhinitis (AR). Within the 40 patients assessed, the conjunctival microbial diversity was found to be significantly reduced and different in composition in the group with ARC and AR [146]. Specifically, they found that the phylum of Proteobacteria was the most abundant in the ARC and AR groups. On the genus level, *Pseudomonas* was the most abundant, and *Corynebacterium* was significantly low in the ARC and AR groups compared to the healthy subjects [146]. Low diversity of microorganisms is considered unhealthy and has been related to other chronic diseases. Deng and colleagues indicated that a low diversity of gut microbiomes was correlated with obesity and type 2 diabetes [155].

OSM in patients with allergic disease indicates a significant decrease in diversity and exacerbation of dysbiosis compared to healthy conjunctival flora [156]. Such microbiome alterations emphasize the importance of conducting further research with cutting-edge technologies to improve our understanding and develop optimal treatment options for patients suffering from AC.

## 6. Impact of Antibiotic Treatments on the Ocular Surface Microbiome

Antibiotics play a pivotal role in altering the ocular surface microbiome. Although antibiotics can treat and prevent infectious and inflammatory conditions, they can also lead to long-term, negative side effects, including increased corneal bacterial presence of certain bacterial strains [35,157,158] or enhancing the virulence of a colonizing pathogen [159]. Moreover, the long-term usage of antibiotics can lead to increasing resistance of the microbiome and promote the spread of antibiotic resistance genes (ARGs), which can lead to damaging consequences [160,161].

Antibiotic usage can cause profound changes in the microbiome within the entire body, including the ocular surface [162,163,164]. A few studies have explored the impact of antibiotics on the ocular surface microbiome. Based on culture-based methods, Ozkan et al. found that the number and variety of microbiota decreased from the use of prophylactic topical tobramycin after continuous use for three months [165]. Moreover, Ono et al., found that it took six months to restore the natural ocular flora after a one-month use of levofloxacin after patients had cataract surgery [166]. Although culture-based methods have been widely used previously, these methods severely underestimate the plethora of microbiome data [52,54]. However, more recent molecular techniques, such as 16S ribosomal RNA sequencing, have discovered a rich and diverse ocular surface flora [66,141,167]. Hotta et al. found that the ocular surface bacterial composition was disrupted and reduced in diversity 2 weeks after stopping gatifloxacin post-cataract surgery [168]. Even after cessation of antibiotic treatment, the ocular surface microbiome was impacted and took 4 weeks to recover to its original state [168]. Another study using 16S rRNA sequencing found that ocular surface species diversity, community structure, and composition were severely affected after exposure to ceftazidime, tobramycin, and vancomycin combination treatment, and it took 30 days to restore after cessation [35]. In conclusion, antibiotic use can severely impact the ocular surface microbiome’s composition and diversity, but they tend to restore to their original state after treatment is discontinued. These studies highlight the plasticity of the ocular surface microbiome and its ability to self-restore after cessation of antibiotic use.

## 7. Contact Lens Wear Impacts on the OSM

The use of contact lenses is a well-established risk factor for the development of inflammatory conditions including microbial keratitis [5,169,170]. Contact lens usage has been associated with dysbiosis of the OSM leading to an increase in pathogenic organisms resulting in higher rates of ocular infection [5,71,170,171]. Wearing contact lenses can disrupt the ocular surface through mechanical friction, which may disturb the delicate balance of the OSM and promote microbial dysbiosis [171,172]. Dysbiosis can also be influenced by the type of contact lenses worn—rigid or soft—as each can impact the epithelial cells of the cornea and conjunctiva [4], thereby contributing to alterations in the ocular surface microbiota [71]. An increased abundance of certain genera—such as *Staphylococcus* and *Pseudomonas* —which are normally part of the ocular surface microbiota (OSM), can predispose individuals to various ocular conditions, including corneal infiltrative events, bacterial keratitis, and corneal ulcers [170]. *Pseudomonas aeruginosa*, in particular, is frequently isolated in cases of bacterial keratitis. The presence of a healthy OSM plays a crucial role in reinforcing the ocular immune barrier and regulating susceptibility to infections caused by *P. aeruginosa* [5].

Multiple studies on contact lens wearers have shown that their OSM differs from that of non-contact lens wearers, with a composition more closely resembling skin microbiota—characterized by increased levels of *Pseudomonas* and a reduction in commensal bacteria normally present in the OSM [5,16,77,172]. The increased presence of skin-associated bacteria within the OSM of contact lens wearers suggests that contact lenses may serve as a vehicle for transferring skin microbes to the eye, potentially disrupting the natural composition of the OSM [16]. The presence of *Corynebacterium* spp. and *Staphylococcus epidermidis* on contact lenses is likely to result in the development of contact lens peripheral ulcers in contact lens wearers. In contrast, the presence of Gram-negative bacteria on the contact lens surface can make the wearer prone to developing contact lens-induced acute red eye (CLARE) [14]. In one study involving contact lens wearers, levels of *Staphylococcus*, *Streptococcus*, *Corynebacterium*, and *Haemophilus* were found to be reduced, while there was a relative increase in skin-associated bacteria such as *Pseudomonas*, *Acinetobacter*, *Methylobacterium*, and *Lactobacillus* [16]. These findings were supported by another study, which also reported higher levels of *Methylobacterium* spp., *Acinetobacter* spp., and *Pseudomonas* spp. in contact lens users, along with decreased abundance of *Haemophilus* spp., coagulase-negative *Staphylococci*, *Streptococcus* spp., and *Corynebacterium* spp. [173]. In another study, there was an overabundance of *Enterobacter mori*, *Staphylococcus pasteuri*, and *Achromobacter agilis* [169]. Additionally, *Staphylococcus pasteuri*—a Gram-positive bacterium commonly associated with skin microflora or waterborne contaminants—was identified as the most prevalent organism in contact lens wearers [169]. This shift in microbiota composition is likely influenced by environmental contaminants present on the surface of contact lenses [169]. Another study revealed no significant differences in the bacterial diversity in contact lens wearers, or patients with contact lens-associated bacterial keratitis when compared to the non-contact lens wearing group [171]. Although variations in sampling methods may influence these findings, the results suggest that the commensal microbiota of OSM have the potential to act as opportunistic pathogens under certain conditions [171]. Wearing contact lenses can disrupt the ocular surface by reducing corneal oxygenation, increasing cellular hypoxia, and promoting microabrasions—all of which compromise the corneal barrier and heighten susceptibility to pathogenic invasion [169]. Additionally, environmental and behavioral factors such as dry eye syndrome, antibiotic use, personal hygiene habits, and improper contact lens practices can further alter the OSM and promote colonization by pathogenic microorganisms [5]. Both Gram-negative and Gram-positive bacteria are capable of causing microbial keratitis, and many of these pathogens can adhere to contact lens surfaces, facilitating biofilm formation and increasing resistance to clearance and treatment [169].

## 8. Modalities for Combating Gut Dysbiosis

Modifying the gut microbiome by normalizing its microbiota may pave the path for novel therapeutics to manage various ocular diseases [116]. There are three common methods for altering gut microbiota: fecal microbiota transplantation (FMT), the application of probiotics (potentially beneficial microorganisms), and the use of prebiotics (nondigestible food ingredients for boosting specific populations of microorganisms) [174,175]. Probiotics can protect against pathogens due to their antimicrobial properties, whereas prebiotics can promote the growth of good bacteria [176]. On the other hand, FMT can directly change the recipient’s microbial composition by transferring gastrointestinal microbiota from a healthy donor, thereby restoring microbial diversity [177].

**Figure 2:**
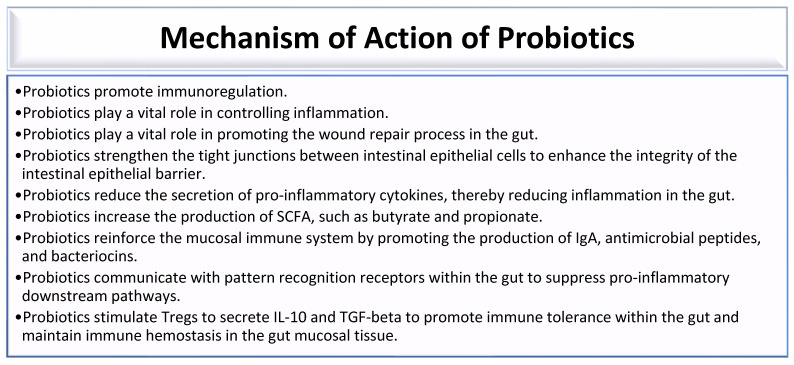
Mechanisms of action of probiotics [12,30,179,183,184].

Probiotics are live microorganisms that combat gut dysbiosis and possess health-promoting benefits. Some bacterial species of genera, which include *Lactobacillus*, *Bacillus*, *Escherichia*, *Enterococcus*, *Pediococcus*, *Streptococcus*, *Leuconostoc*, *and Bifidobacterium,* possess probiotic properties that confer health benefits to individuals with gut dysbiosis [178]. Probiotics promote immunoregulation by ensuring a balanced immune response (Figure 2) [12]. Probiotics play a vital role in controlling inflammation and promoting the wound repair process in the gut (Figure 2) [12,30]. They strengthen the tight junction between intestinal epithelial cells to enhance the integrity of the intestinal epithelial barrier (Figure 2) [12,30,179]. Thus, probiotics prevent an increase in intestinal transepithelial permeability, thereby preventing the leakage of bacteria and toxins into the systemic circulation. This is made possible by fortifying the intestinal barrier, which is maintained by tight junctions between individual intestinal epithelial cells [12,180]. Some probiotic bacteria produce bacteriocins that inhibit the growth of pathogenic bacteria [12,30]. Bacteriocins produced by some ocular surface bacterial microbiota can inhibit the growth of other microbes [12]. Lactic acid bacteria, such as *Lactobacillus* species, *Streptococcus* species, *Enterococcus* species, and *Lactococcus* species, produce bacteriocins that exhibit antibacterial activity against both Gram-positive and Gram-negative bacteria. These bacteriocins are effective against various pathogens, including *Staphylococcus aureus*, *Pseudomonas aeruginosa*, *Salmonella typhi*, *Shigella flexneri*, *Listeria monocytogenes*, *pathogenic Escherichia coli*, and *Clostridium botulinum* [181,182]. Thus, Lactobacillus, Enterococcus, Streptococcus, and other bacteria that reside in the gut are bacteriocin-producing bacteria that facilitate the destruction of pathogenic bacteria in the gut [181]. Probiotics reduce the secretion of pro-inflammatory cytokines, thereby reducing inflammation in the gut (Figure 2) [30]. Probiotics increase the production of SCFA, such as butyrate and propionate (Figure 2) [30,183]. Probiotics containing Lactobacillus casei, Lactobacillus acidophilus, and Streptococcus thermophilus promote the production of IgA-secreting plasma cells [184]. Thus, probiotics reinforce the mucosal immune system by promoting the production of IgA, antimicrobial peptides, and bacteriocins (Figure 2) [30,179]. Probiotics communicate with pattern recognition receptors within the gut to suppress pro-inflammatory downstream pathways (Figure 1) [30,179]. Probiotics stimulate Treg cells to secrete IL-10 and TGF-beta to promote immune tolerance within the gut and maintain immune hemostasis in the gut mucosal tissue (Figure 2) [30,184].

Tavakoli et al. assessed the benefits of probiotics and prebiotics in a randomized controlled trial of 41 participants with dry eye disease and found a significant improvement in the signs and symptoms of dry eyes using the Ocular Surface Disease Index (OSDI) [175]. Another study found that the probiotics of hydrolyzed casein formula, which consists of probiotic *Lactobacillus rhamnosus GG*, reduced allergic manifestations and increased immune tolerance in children with cow’s milk allergy [185]. Agreeably, a study conducted by Pastor-Villaescusa et al. discovered a significantly lower incidence of conjunctivitis in infants whose nursing mothers received a probiotic treatment of *Lactobacillus fermentum* CECT5716 (Lc40) [186].

These studies show promising results of a novel field of research and solidify the link between gut and oral microbiome dysbiosis and the ocular surface. However, there is a need for more comprehensive studies that can aid researchers and clinicians in finding a practical and effective application of such therapeutics. These treatment options may be the answer to relieving patients’ ocular disease symptoms and improving their quality of life.

## 9. Conclusions

The ocular surface microbiome is rich, abundant, and diverse in composition and plays a crucial role in maintaining homeostasis and preventing pathogens from invading. In addition to the OSM, the health and dysbiosis of the gut and oral microbiome impacts the progression of ocular diseases. Environmental conditions, ocular diseases, antibiotic use, age, gender, personal habits, and contact lens wear can also impact the composition and diversity of the ocular surface microbiome. Thus, preserving the epithelial barrier function of the ocular surface, dampening the inflammatory process, and maintaining immune tolerance to the ocular surface microbiome are essential for a disease-free state.

Modern technological advances, such as next-generation sequencing and high-throughput sequencing technology, are promising in allowing us to accurately find the makeup of the commensal microbes that form the OSM. Understanding the OSM and its close relationship to the gut and oral microbiome is a crucial step towards accurate diagnosis and appropriate management options. The advent of using probiotics, prebiotics, and FMT is an exciting and unique method of treatment for various ocular diseases, and eye care providers can add to as a treatment modality for severe ocular surface disease patients.

## Figures and Tables

**Table 1:** Current composition of the ocular surface microbiome.

Bacteria	Virus	Fungi
Corynebacterium Acinetobacter Staphylococcus Cutibacterium Streptococcus Pseudomonas Anaerococcus Finegoldia Simonsiella Veillonella Millisia	Multiple Sclerosis-associated retrovirus (MSRV) Human Endogenous Retrovirus K (HERV-K) Torque Teno Virus (TTV)	Malassezia Rhodotorula Davidiella Aspergillus Alternaria Fusarium Curvularia Penicillium Helminthosporium Setosphaeria Haematonectria Candida Choriomyces Claedesporium Saccharomyces Hormodendrum

## Data Availability

No new data were created or analyzed in this study. Data sharing is not applicable to this article.
